# A study on catalytic and non-catalytic sites of H5N1 and H1N1 neuraminidase as the target for chalcone inhibitors

**DOI:** 10.1186/s13765-021-00639-w

**Published:** 2021-09-17

**Authors:** Pandu Hariyono, Jasvidianto Chriza Kotta, Christophorus Fideluno Adhipandito, Eko Aprilianto, Evan Julian Candaya, Habibah A. Wahab, Maywan Hariono

**Affiliations:** 1 Faculty of Pharmacy, Sanata Dharma University, Campus III, Paingan, Maguwoharjo, Depok, Sleman, 55282 Yogyakarta Indonesia; 2 Faculty of Biomedical Engineering, Taipei Medical University, Wuxing Street No. 250, Xinyi District, Taipei City, 110 Taiwan; 3 PT. Dankos Farma, Jalan Rawagatel Blok IIIS Kav 35-39, Jatinegara, Cakung, Jakarta Timur, 13930 DKI Jakarta Indonesia; 4 Apotek Kimia Farma Sempidi Unit Bisnis Nusa Dua, Jalan Raya Sempidi No. 12, Mengwi, Badung, 80351 Bali Indonesia; 5 Pharmaceutical Technology Department, School of Pharmaceutical Sciences, Universiti Sains Malaysia, Minden, 11800 Pulau Pinang Malaysia

**Keywords:** Neuraminidase, Non-catalytic site, Chalcone, Influenza, H5N1, H1N1

## Abstract

The H1N1 pandemic in 2009 and the H5N1 outbreak in 2005 have shocked the world as millions of people were infected and hundreds of thousands died due to the infections by the influenza virus. Oseltamivir, the most common drug to block the viral life cycle by inhibiting neuraminidase (NA) enzyme, has been less effective in some resistant cases due to the virus mutation. Presently, the binding of 10 chalcone derivatives towards H5N1 and H1N1 NAs in the non-catalytic and catalytic sites was studied using molecular docking. The in silico study was also conducted for its drug-like likeness such as Lipinski Rule, mutagenicity, toxicity and pharmacokinetic profiles. The result demonstrates that two chalcones (**1c** and **2b**) have the potential for future NA inhibitor development. Compound **1c** inhibits H5N1 NA and H1N1 NA with IC_50_ of 27.63 µM and 28.11 µM, respectively, whereas compound **2b** inhibits NAs with IC_50_ of 87.54 µM and 73.17 µM for H5N1 and H1N1, respectively. The in silico drug-like likeness prediction reveals that **1c** is 62% better than **2b** (58%) in meeting the criteria. The results suggested that **1c** and **2b** have potencies to be developed as non-competitive inhibitors of neuraminidase for the future development of anti-influenza drugs.

**Supplementary Information:**

The online version contains supplementary material available at 10.1186/s13765-021-00639-w.

## Introduction

The novel Severe Acute Respiratory Syndrome (SARS) coronavirus, SARS-CoV-2, which was first discovered in December 2019 in Wuhan, China, infected approximately 64,000 people with about 1400 deaths announced at that time, mainly in China [1]. At almost the same time, the country was also reportedly dealing with an outbreak of the deadly H5N1 avian influenza in Hunan, an area that borders Hubei, the province where the new coronavirus emerged, according to the South China Morning Post [2]. As of February 1, 2020, local authorities had culled 17,828 poultries after the H5N1 outbreak, according to China’s Ministry of Agriculture and Rural Affairs statement [3]. Fortunately, there are no reported human cases of this H5N1 avian flu, although the transmission of the disease to humans had been reported since the first infection to humans recorded in 2003 [4].

Since the H5N1 outbreak in 2005, the world has been prepared for the impending influenza pandemic, and in 2009, H1N1 strains emerged and threatened humanity with its spread in more than 70 countries [5]. However, the severity of the 2009 H1N1 pandemic was modest compared to the H5N1 outbreak. H5N1 strain is considered particularly dangerous because of its human fatality rate of over 50% to date and because of the risk that the virus may develop the ability to pass efficiently between humans. As of October 2020, the World Health Organization (WHO) reported a total of 861 confirmed human cases which resulted in the deaths of 455 people since 2003 [6]. Thus, these instances in history warrant an enhanced pandemic preparedness, especially in this COVID-19 season. Although there have been no detected complications across the population in either seasonal or pandemic H1N1 influenza, the elderly patient has a higher risk for hospitalization and mortality during the seasonal H1N1 influenza [7]. In particular, patients having specific chronic conditions such as heart disorder, lung disease, hyperglycemia, renal failure, rheumatics, dementia, and thromboembolism are at high risk of influenza complications [8, 9].

The influenza virus is known to be rapidly mutated in which oral anti-influenza, oseltamivir (Tamiflu), has been less potent to the H1N1 infection [10]. Therefore, a study on finding a new anti-influenza agent should be continuously encouraged. Neuraminidase is the most common targeted protein in the therapy of influenza, although hemaglutinin and M2 ion channels were also studied [11]. NA has an important role in cleaving new virions from the infected cell [12]. Once it is blocked, the new virus could not spread over other organs [13]. Reverting to the problem of virus resistance, a new chemical that has not been recognized by the virus is highly desired [14].

A series of chalcone compounds have been reported to demonstrate anti-neuraminidase activity [15] together with their quantitative-structure activity relationships (QSARs) study. The QSAR model suggested that a functional group having electronic characters would increase the activity of the chalcone. In contrast, the steric effect would decrease its activity against H1N1 NA. Other chalcones having anti-NA activities were isolated from *Glycyrrhiza uralensis* [16], *Erythrina addisoniae* [17], *Glycyrrhiza inflate* [18], *Polygala karensium* [18], and *Angelica keiskei* [19] further supporting the claim of chalcones as potential NA inhibitors.

However, upon inspection of the chemical structure, chalcone might not be a favorable scaffold for NA inhibitors which usually belong to the shikimic acid structure. The shikimic acid inhibitors such as oseltamivir and zanamivir bind to the catalytic site of NA, which is a small pocket surrounded by basic sub-pocket, acidic sub-pocket as well as hydrophobic sub-pocket alongside its conserved amino acid residues [20]. Chalcone is a phenyl ring extended by an *α*,*β*-unsaturated carbonyl with various alkyl or aryl moieties next to the carbonyl group, making it too bulky to fit into the NA catalytic site [21]. A chalcone compound, namely 2-amino-5-[3-[4-[(E)-4-chloro-3-oxobut-1-enyl]anilino]propyl]-4-methyl-1H-pyrimidin-6-one (NSC89853) is reported to show H5N1 NA inhibition from the screening of 20 compounds from NCI database. This inhibition may be due to the binding to the protein with an alternative mode, that is different from the known drugs (oseltamivir or zanamivir). Although the potency of such a compound was not as strong as the known drugs, it may overcome the drug i.e. H274Y for the N1 protein [22].

**Fig. 1:**
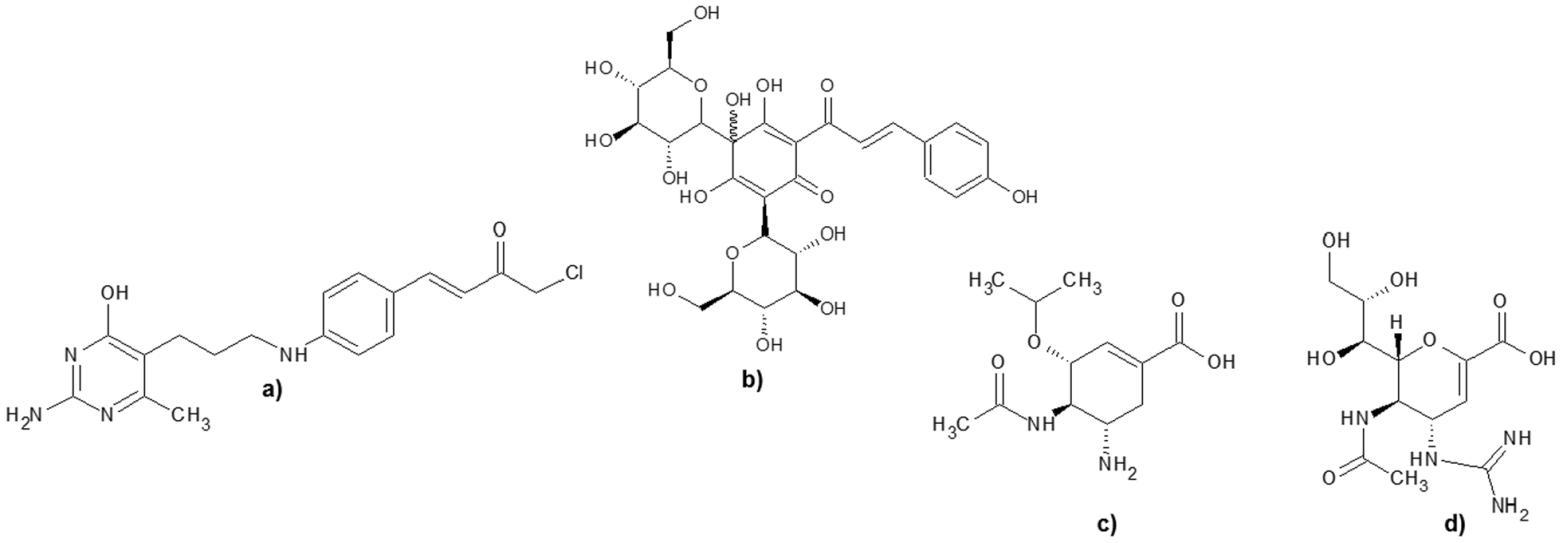
The structure of **a** NSC89853, and **b** SA, which are suggested to be non-catalytic site inhibitors of H5N1 and H1N1, respectively, whereas **c** oseltamivir, and **d** zanamivir are known NA inhibitors targetting the catalytic sites

In one of the more recent research, safflomin A (SA) was reported to show inhibition against NAs from H1N1 and H3N2 influenza viruses. The antiviral assay using MDCK cells which was infected by H1N1 and H3N2 influenza virus exhibited the synergistic effect of SA with oseltamivir in viral cell proliferation. The kinetics study of SA demonstrated a non-competitive inhibition against N1 and N2 NA. Furthermore, a molecular docking study predicted that SA interacted with N1 and N2 NA at the non-catalytic site. These results suggested that SA, which has a chalcone backbone, may serve as a potential therapeutic option to the currently available anti-influenza agents to overcome the drug resistance [23]. The structure of NSC89853, SA, oseltamivir and zanamivir are presented in Fig. 1.

In this present study, we examined 10 chalcone derivatives for their predicted binding affinities into the non-catalytic site of H1N1 and H5N1 NA using a molecular docking study. The non-catalytic location is at the back of the NA’s catalytic site within loop 150. Molecular docking of chalcones into the NA catalytic site was also conducted to compare their predicted binding affinities with the ones in the non-catalytic site. Further in silico studies to predict the drug-like likeness were also carried out employing Lipinski Rule, pharmacokinetic profile and toxicity prediction. The study was then followed by testing for their biological activities against H5N1 and H1N1 NA using in vitro MUNANA assay. In addition, the safety index was also calculated by studying the cytotoxicity effect of the most active compound toward a normal cell. Two chalcones demonstrated potential activity as neuraminidase of H5N1 and H1N1 inhibitors, respectively, with low toxicities to the normal cell lines and predicted to have considerably good drug-like structures.

## Results

### Molecular docking

**Fig. 2:**
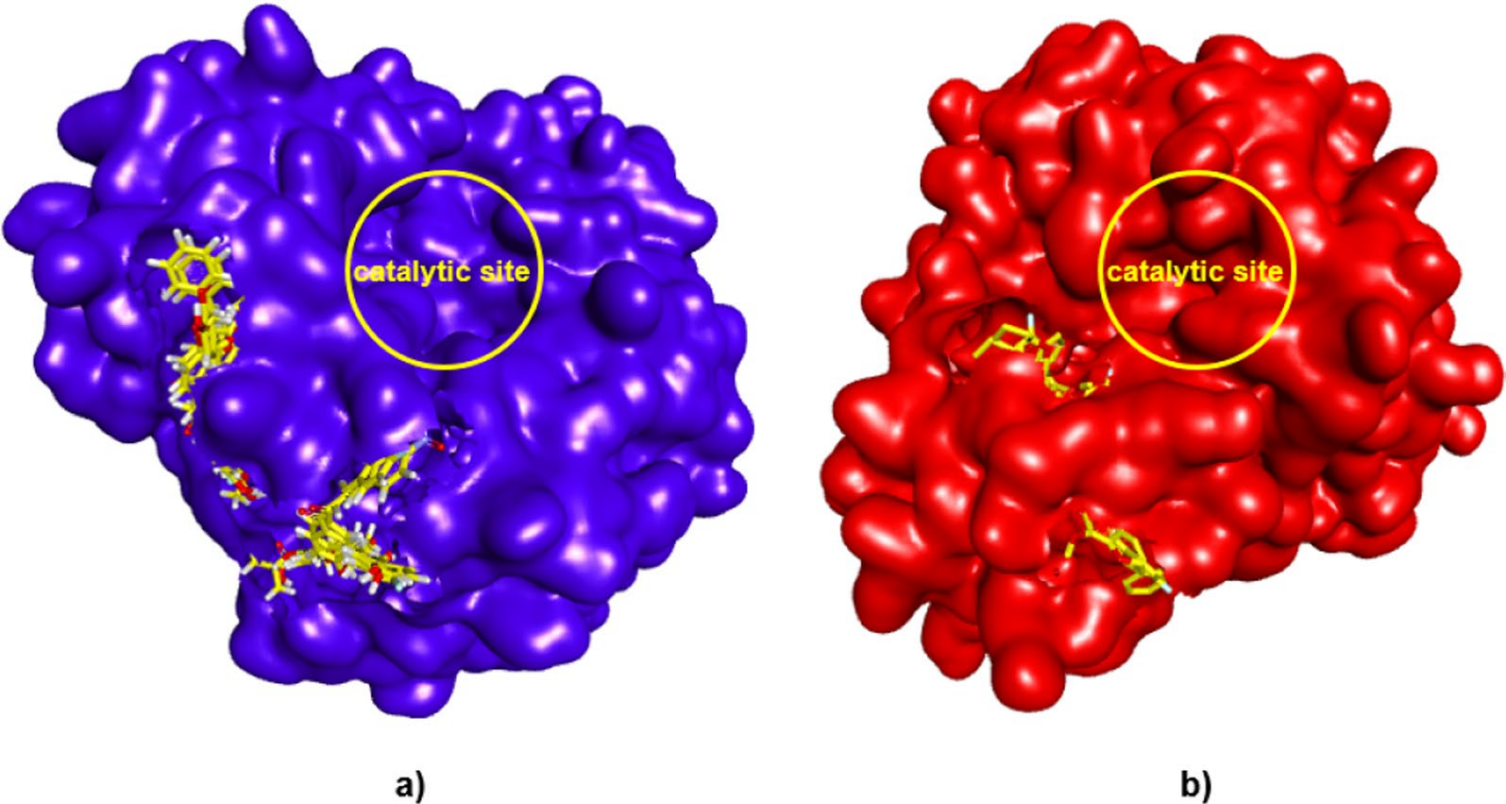
The superimposition of 10 chalcone derivatives (yellow sticks) in the non-catalytic site of **a** H1N1 and **b** H5N1 NA. The NA is presented in the surface model and the ligands are in the stick models, docked in the behind of the catalytic site (yellow circles)

The ten chalcone derivatives have been predicted for their molecular interactions using docking study as presented in Table 1. The ΔG_bind_ ranged at − 6.12 to − 7.97 kcal/mol and − 6.34 to − 8.31 kcal/mol at H1N1 NA and H5N1 NA non-catalytic sites, respectively. These results describe that chalcone has a potency from a low to moderate micromolar activity (K_i_ 32.71 to 1.44 µM at H1N1 NA; and K_i_ 22.58 to 0.82 µM at H5N1 NA) to bind at this non-catalytic site and disrupt the enzyme activity. The superimposition of ten chalcone derivatives in the non-catalytic site of H1N1 and H5N1 NAs is illustrated in Fig. 2.

**Table 1:** The docking results of 10 chalcone derivatives in the non-catalytic site of H1N1 and H5N1 NA

Compounds	H1N1 NA			H5N1 NA		
	ΔG_bind_ (kcal/mol)	Residues	Predicted K_i_	ΔG_bind_ (kcal/mol)	Residues	Predicted K_i_
**1a**	− 6.48	ARG118, ARG156, ARG430	17.68 μM	− 7.12	ARG118, ARG156, ARG430	6.02 μM
**2a**	− 6.92	SER196, VAL203, LYS207	8.50 μM	− 8.31	LEU134, ARG156, ARG430	815.58 nM
**3a**	− 6.68	SER196, ARG207	12.66 μM	− 8.18	VAL116, ARG118, LEU134, THR135, ARG156, ARG430	1.00 μM
**1b**	− 6.12	PHE174, SER196	32.71 μM	− 6.34	ARG118, ARG430, THR439	22.58 μM
**2b**	− 6.18	LYS150, VAL177	29.34 μM	− 7.32	VAL116, GLN136, GLY147, VAL149, HIS155	4.33 μM
**3b**	− 7.15	VAL116, ALA138, HIS144, ILE149	5.74 μM	− 7.58	VAL116, ARG118, VAL149, ASP151, THR439	2.80 μM
**4b**	− 7.97	LYS150, VAL177, SER196, ASP199, VAL205	1.44 μM	− 7.89	ARG118, ARG156, ARG430, PRO431	1.65 μM
**1c**	− 6.72	HIS144, ILE149, THR438	11.82 μM	− 6.53	VAL116, ILE117, ARG118, LEU134, ARG430	18.30 μM
**2c**	− 6.02	LYS143, HIS144	38.83 μM	− 7.55	ARG118, HIS144, VAL149, ASP151, HIS155, THR439	2.95 μM
**3c**	− 6.27	HIS144, TYR155	25.18 μM	− 7.94	VAL116, ARG118, LEU134, ARG156, ARG430, PRO431	1.52 μM

To compare the binding mode of the ligands to the common site, a molecular docking study was also conducted into the H1N1 and H5N1 NA catalytic sites. The parameterization of molecular docking was controlled by re-docking oseltamivir-triazole into the H1N1 NA and oseltamivir into the H5N1 NA catalytic site. This resulted in RMSD 1.02 Å of the most populated cluster (85%) with ΔG_bind_ − 9.35 kcal/mol at H1N1 NA, whereas the RMSD of oseltamivir at H5N1 NA active site was 1.9 Å of the most populated cluster (74%) with the lowest energy (ΔG_bind_ − 7.52 kcal/mol). This defines that the parameter is accepted for further docking of chalcone compounds. The control docking pose of ligands to their individual NA which are overlapped with its initial pose is presented in Additional file 1: Figure S1.

**Fig. 3:**
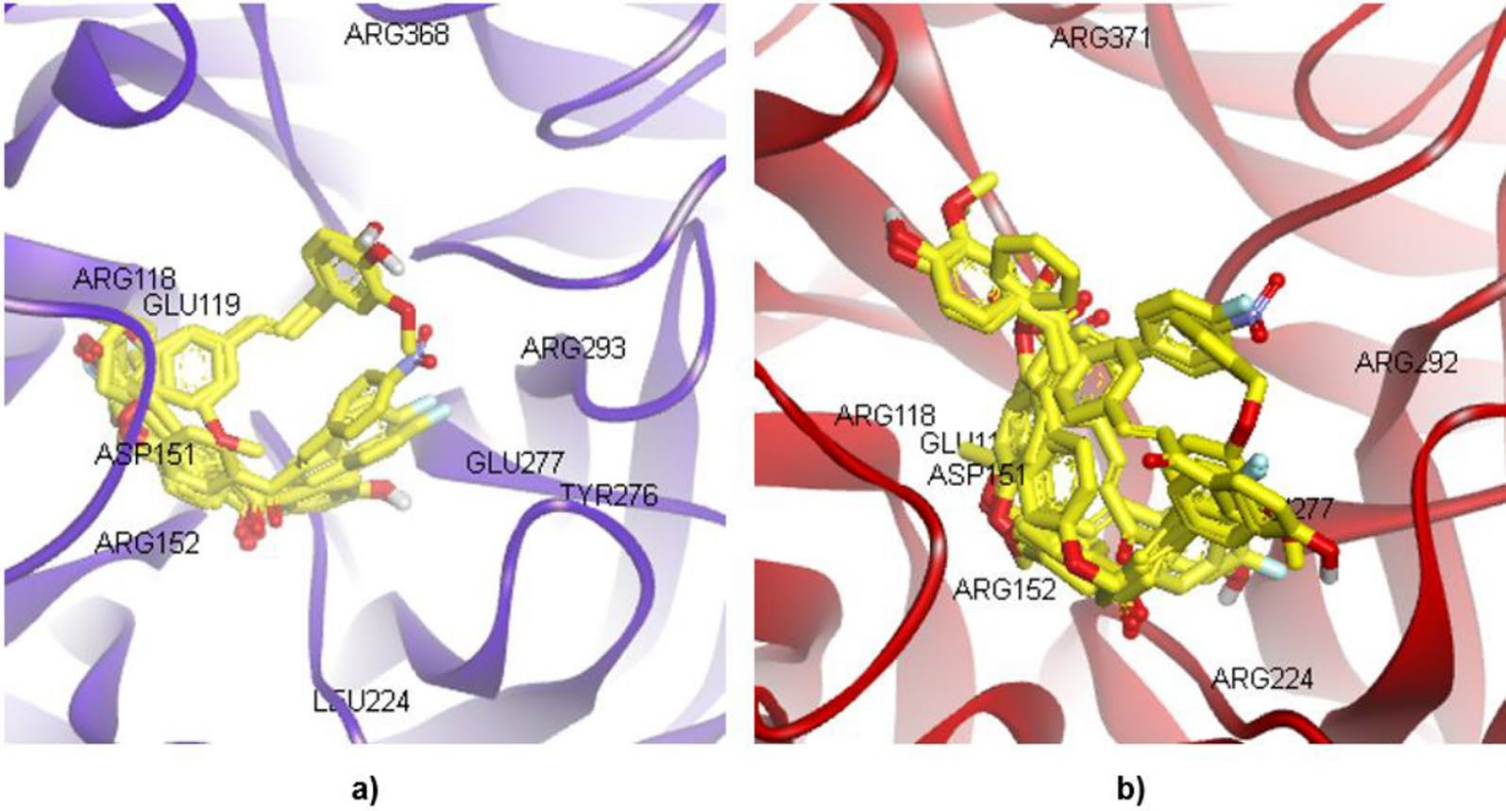
The superimposition of 10 chalcone derivatives (yellow sticks) in the active site of **a** H1N1 NA (blue) and **b** H5N1 NA (red) in the ribbon model

The control docking pose closely interacts with the conserved amino acid residues such as ARG118, ASP151, TRP179, ARG293, ARG368 and TYR402 via hydrogen bondings. The parameter of that control docking is then used to dock 10 chalcone derivatives into both H1N1 and H5N1 NA active site binding pockets. The docking results of 10 chalcone derivatives are presented in Table 2 represented by the ΔG_bind_, interacting residues and the predicted K_i_. The docking into H1N1 results in ΔG_bind_ − 6.37 to − 8.34 kcal/mol, whereas it is ΔG_bind_ − 5.05 to − 6.93 kcal/mol into H5N1 for the overlapped-docked pose which is presented in Fig. 3. The calculated energies of docked chalcones into the H1N1 catalytic site are likely lower than that of H1N1 at the non-catalytic one. In contrast, the chalcones docked into the H5N1 have lower free energy of binding in the non-catalytic than in the catalytic sites. However, except for compounds **1a**, **2a** and **3a,** its long alkyl chain escaped from the catalytic site, indicating their non-fit binding into both H1N1 and H5N1 NA’s catalytic sites. Table 2 tabulated the docking results of the 10 compounds in the active site of H1N1, H5N1 NA, and their superposition as illustrated in Fig. 3.

**Table 2:** The docking results of 10 chalcone derivatives in the catalytic site of H1N1 and H5N1 NA

Compounds	H1N1 NA			H5N1 NA		
	ΔG_bind_ (kcal/mol)	Residues	Predicted K_i_	ΔG_bind_ (kcal/mol)	Residues	Predicted K_i_
**1a**	− 6.37	ARG152, ARG156, ARG225, GLU277, GLU278	21.55 μM	− 5.69	GLU119, ARG152, ARG156, ILE222, SER246	67.9 μM
**2a**	− 7.71	ARG152, ARG225, THR226, GLU278	2.22 μM	− 6.66	ARG118, ARG152, ILE222, GLY244, SER246, ARG292, ARG371	13.07 μM
**3a**	− 7.39	GLU119, LYS150, ASP151, ASP152, ARG225, GLU277, GLU278	3.86 μM	− 6.07	ARG118, ARG152, ARG224 THR225, GLU277, TYR347, ARG371, TYR406	35.7 μM
**1b**	− 7.36	ARG118, ARG152, TRP179, GLU277, GLU278, ARG293	4.03 μM	− 6.26	ARG152, ARG156, ILE222, SER246, TYR406	25.86 μM
**2b**	− 8.10	ARG118, ARG152, TRP179, GLU277, ARG293, ASN295	1.15 μM	− 6.52	ARG118, ASP151, ARG156, ARG292, TYR347, ARG371	16.63 μM
**3b**	− 8.34	ARG118, ARG152, ARG156, TRP179, GLU278, ARG293, ASN295	0.772 μM	− 6.41	ARG118, ASP151, ARG156, ARG224, GLU276, ARG277, ARG371	20.05 μM
**4b**	− 7.13	ARG118, LEU134, LYS150, ASP151, ARG152, ARG293, ASN344	5.91 μM	− 6.88	ARG118, ASP151, ARG156, GLU277, ARG292, TYR347, ARG371	9.04 μM
**1c**	− 6.57	GLU119, ARG152, ARG156, GLU277, GLU278	15.34 μM	− 5.05	ARG118, ASP151, ARG152, TRP178, ARG224, TYR347, ARG371, ARG430, PRO431	200.37 μM
**2c**	− 7.49	GLU119, LYS150, ASP151, ARG152, TRP179, ARG293, ASN344, ARG368, TYR402	3.25 μM	− 6.86	ARG118, GLU119, LEU134, ASP151, ARG152, ARG156, ARG224, GLU227, TYR347, ARG371, ILE427, ARG430, PRO431	9.41 μM
**3c**	− 7.75	ARG118, GLU119, LYS150, ASP151, ARG152, TRP179, ARG293, ASN344, ARG368, TYR402	2.09 μM	− 6.93	GLU119, ARG152, ARG156, ILE222, ARG224, GLY244	8.32 μM

### Lipinski rule

According to the Lipinski Rule, an ideal drug should have a molecular weight (MW) which is less than 500 Da, < 5 log P, ≤ 5 Hydrogen Bond Donor (HBD), ≤ 10 Hydrogen Bond Acceptor (HBA), ≤ 10 rotatable bonds, and ≤ 140 Å surface area [24, 25]. The potency of the compound to be drug is commonly expressed by IC_50_, which is affected by its MW. The more potent drug should have a higher MW to minimize its dose in performing a pharmacological activity. Nevertheless, the MW should not be higher than 500 considering the drug absorption via the intestinal membrane [24].

The log P is the concentration of the compound in *n*-octanol divided by its concentration in water. Therefore, it reflects the balance of the compound’s solubility in water during oral dissolution steps with its oral bioavailability in the blood system [24, 25]. The ideal log P < 5 indicates that the compounds are well soluble in the body fluid as well as absorbed through the gastrointestinal cell membrane, which then be transferred into the blood vascular. On the other hand, the number of HBD or HBA is associated with polarity to interact with water during the dissolution process as well as their molecular interaction via hydrogen bond with its receptor [24]. The rotatable bonds may influence their stability during ADME (absorption, distribution, metabolism, and excretion) and the receptor binding. Thus, the less flexible the chain in the molecule, the more stable the drug is to perform its activity [25]. The Polar Surface Area (PSA) is related to the permeability of drugs across the cell membrane in which the higher the PSA, the poorer the cell permeability (oral bioavailability) is [25].

In general, the results show that all chalcones meet the MW, the number of HBD, HBA and the rotatable bonds requirements. However, compounds **2b** and **3b** slightly deviate on the log P limit, whereas compounds **2c**, **3c** and **4b** do not fulfill the limit of the surface area requirement. Table 3 presents the chalcones with their Lipinski Rule profiles.

**Table 3:** The drug-like likeness profile of 10 chalcones was studied as NA inhibitors

Ligands	MW	log P	HBD	HBA	Rotatable bonds	Surface area
1a	242.249	3.4274	1	2	3	104.015
1b	284.330	4.5090	0	2	5	123.429
1c	312.365	4.0857	1	4	7	135.536
2a	269.256	3.1965	1	4	4	114.502
2b	310.368	5.0432	0	2	5	135.153
2c	390.435	4.8845	1	5	8	169.342
3a	268.268	2.9865	2	3	4	115.170
3b	318.347	5.5141	0	2	5	139.391
3c	360.409	4.8759	1	4	7	157.864
4b	337.375	4.8123	0	4	6	145.641

### Mutagenicity and toxicity profiles

The in silico toxicity predictions have become a routine before processing compounds to be drug candidates. In this study, we also predicted the mutagenicity and toxicity profiles of the 10 chalcones. Generally, a compound’s mutagenic properties are usually confirmed using the AMES test [26, 27]. The human Maximum Tolerated Dose (hMTD) is acceptable when the predicted toxic dose threshold in humans is more than 0.477. The potassium channels that mediate the cardiac repolarization in humans are represented by the values of hERG I and II. Hence, inhibiting this kind of protein would cause a long QT syndrome development that might lead to a fatal arrhythmia. The in vivo toxicity is often expressed by LD_50_ value which can be defined as the required dose given to cause 50% death of a group of rats in evaluating a compound acute toxicity represented by ORAT (log LD_50_ value) prediction.

LOAEL (lowest-observed-adverse-effect level) associates with the compound’s lowest concentration which causes an adverse effect in human physiology. This is indicated by the alteration of morphology, function, growth, or development. The safety of a compound is higher as the LOAEL value increases. The liver injury commonly reflects the drug hepatotoxic properties, whereas the potential dermal adverse effects are determined by skin sensitization properties. The value of toxic endpoints, however, is measured by *T. pyriformis* and minnow toxicity [28].

Nowadays, the drugs’ safety to the environment is a concern. Low environmental damage is demonstrated by the values of *T. pyriformis* and minnow toxicity which are to be respectively higher than 0.5 and − 0.3. Based on the AMES test, two compounds i.e. **2a** and **4b** are predicted not to be toxic, while **4b** might have the possibility to be hepatotoxic. No chalcones inhibit the hERG I protein, but **1c**, **2b**, **2c** and **3c** inhibit hERG II. Most of the chalcones have a low maximum dose which is weakly tolerated in humans, except for **2a**, **2b** and **4b**. In addition, all chalcones exhibit no skin sensitization. The oral rat acute toxicity LD_50_ can be classified as very toxic (≤ 5 mg/kg), toxic or moderately toxic (> 5 to < 500 mg/kg), harmful or slightly toxic (> 500 to < 2000 mg/kg), and non-toxic (> 2000 mg/kg) [29]. All compounds are predicted to be potentially less toxic with LD_50_ ranging from 194.01 to 399 mg/kg. Oral rat LOAEL value shows that the ingestion chronic toxicity is relative to the limit of concentration and length of exposure, however, LOAEL does not solely represent drug safety accurately [30, 31]. This prediction showed that compounds **2a**, **2c**, **3a** and **4b** have lower values than the other compounds, which can be interpreted as they are able to cause any observable chronic toxicity at the lower amount and/or shorter time of exposure. Several compounds (**2c**, **3a**, **3b** and **3c**) demonstrate *T. Pyriformis* toxicity potency, whereas, based on the minnow toxicity prediction, compounds **1a**, **2a**, **3a** and **4b** are deemed to be safe. Table 4 presents the AMES test results of the chalcones for mutagenicity prediction along with other toxicity profiles*.*

**Table 4:** The AMES test result of the chalcones for mutagenicity prediction along with other toxicity profiles

Ligands	AMES toxicity	hMTD	hERG I inhibitor	hERG II inhibitor	ORAT (log LD_50_)	ORCT (log LOAEL)	Hepatotoxicity	Skin Sensitisation	*T. Pyriformis* toxicity	Minnow toxicity
1a	No	0.646	No	No	2.228	2.203	No	No	1.380	0.262
1b	No	0.830	No	No	2.324	2.321	No	No	1.115	− 1.141
1c	No	0.611	No	Yes	2.318	2.044	No	No	1.207	− 0.820
2a	Yes	0.039	No	No	2.485	1.267	No	No	1.231	− 0.229
2b	No	0.429	No	Yes	2.360	2.117	No	No	1.664	− 0.595
2c	No	0.521	No	Yes	2.577	1.164	No	No	0.353	− 1.641
3a	No	0.638	No	No	2.233	1.715	No	No	0.286	0.520
3b	No	0.669	No	Yes	2.519	2.330	No	No	0.421	− 1.293
3c	No	0.502	No	Yes	2.524	2.125	No	No	0.419	− 2.152
4b	Yes	0.039	No	No	2.601	1.231	Yes	No	1.207	− 0.232

### Pharmacokinetic profiles

Table 5 demonstrates that all chalcones are well absorbed through the human intestinal with near values to 100% into the blood system. In general, the water solubility of all chalcones are most likely poor as their log S value are lower than − 4, except for **3a** which has a log S value of − 2.601; indicating that it may dissolve readily in the dissolution step. Furthermore, oral absorption prediction using Caco2 cell model [32] with the required value is higher than 0.90. Therefore, except for **2c** and **4b**, chalcones show good human gastrointestinal absorption. The skin permeability of chalcones is most likely suitable for the transdermal route because the values are approximately − 2.5. A protein transport namely P-glycoprotein (P-gp) is crucial during the pharmacokinetics steps, however, this could have either advantages or disadvantages in therapeutic effect [33]. A compound is supposed to not inhibit P-gp, either P-gp I or P-gp II. In the ideal situation, it should not be acting as P-gp substrate either. According to the prediction, compounds **1c** and **2b** are predicted to inhibit the P-gp I activity, whereas compounds **2c**, **3c**, and **4b** inhibited both P-gp I and P-gp II. In contrast, **2a** acts like the substrate for P-gp, accordingly.

**Table 5:** The absorption profiles of chalcones as predicted by the software

Ligands	Water solubility	Caco2 permeability	Intestinal absorption (human)	Skin permeability	P-glycoprotein substrate	P-glycoprotein I inhibitor	P-glycoprotein II inhibitor
1a	− 4.140	1.426	93.706	− 2.315	No	No	No
1b	− 5.655	1.423	96.342	− 2.434	No	No	No
1c	− 4.798	1.570	93.360	− 2.483	No	Yes	No
2a	− 4.312	0.904	91.606	− 2.627	Yes	No	No
2b	− 5.636	1.636	95.062	− 2.327	No	Yes	No
2c	− 6.726	0.556	93.240	− 2.699	No	Yes	Yes
3a	− 2.601	0.949	100.000	− 2.683	No	No	No
3b	− 6.714	1.684	93.229	− 2.677	No	No	Yes
3c	− 6.342	1.078	95.102	− 2.678	No	Yes	Yes
4b	− 5.522	0.343	93.495	− 2.659	Yes	Yes	Yes

One of the distribution parameters is defined by the number of VDss or volume of distribution at a steady state, which is directly proportional with the amount of drug distributed into tissue; a higher V_D_ indicates a greater amount of tissue distribution. As a rule, VDss should be ≥ − 0.15. Among the 10 chalcones, three compounds (**2c**, **3a**, and **3c**) do not meet these criteria. The fraction unbound (fu) for all chalcones are predicted to be ≤ 0.15, indicating that more fractions of drug molecule are bound to the plasma protein. Besides VDss, the chalcones were also predicted for their ability to cross the brain membrane which is important as the compounds may affect the Central Nervous System (CNS) [34]. Values < − 1 indicate poor Blood–Brain Barrier (BBB) permeability and < − 3 for CNS permeability. In other words, the compound is poorly distributed to the brain and unable to penetrate CNS. From the results, all the 10 compounds should be carefully managed as there is potential for these chalcones to enter the CNS especially for **1a**, **1b**, **2b** and **3b** which can also penetrate BBB. The distribution profiles of the chalcones as predicted by software are presented in Table 6.

**Table 6:** The distribution profile of chalcones as predicted by the software

Ligands	VDss (human)	Fraction unbound (fu) (human)	BBB permeability	CNS permeability
1a	0.015	0.058	0.012	− 1.641
1b	0.106	0.000	0.425	− 1.298
1c	0.087	0.000	− 0.233	− 2.219
2a	0.041	0.000	− 0.328	− 2.123
2b	0.527	0.000	0.398	− 1.465
2c	− 0.476	0.017	− 0.257	− 2.157
3a	− 2.366	0.000	− 0.200	− 2.253
3b	− 0.083	0.035	0.479	− 1.054
3c	− 0.227	0.024	− 0.258	− 2.053
4b	0.638	0.000	− 0.341	− 1.930

Metabolisms are also an important indicator of good drug-like properties. CYP1A2, CYP2C9, CYP2C19, CYP2D6, CYP3A4 are among the cytochrome P450 subfamilies, which play significant roles in drug metabolism [35]. CYP2D6 and CYP3A4 are found in the brain and intestines, respectively, and are most likely responsible to metabolize the drug in their surrounding areas. Furthermore, CYP3A4 also affects oral bioavailability by first-pass metabolism. A compound that binds strongly to these CYPs could be acting as the substrate or inhibitor leading to the lower / higher bioavailability as well as activity of other drugs. This could be potential for the drug causing clinical drug-drug interactions, leading to adverse reactions or therapeutic failures. In this prediction, neither chalcones act as the substrate nor inhibitor for CYP2D6. However, except for **3a**, all compounds act as CYP3A4 substrate, whereas **2c** and **3c** act as this enzyme’s inhibitor. Furthermore, chalcones are likely to inhibit the CYP1A2, CYP2C19, and CYP2C9 presented in Table 7, which should be of concern when the compound is consumed with other drugs.

**Table 7:** The interaction between chalcones with a diverse CYP subfamily

Ligands	CYP2D6 substrate	CYP3A4 substrate	CYP1A2 inhibitor	CYP2C19 inhibitor	CYP2C9 inhibitor	CYP2D6 inhibitor	CYP3A4 inhibitor
1a	No	Yes	Yes	Yes	No	No	No
1b	No	Yes	Yes	Yes	Yes	No	No
1c	No	Yes	Yes	Yes	Yes	No	No
2a	No	Yes	Yes	Yes	Yes	No	No
2b	No	Yes	Yes	Yes	Yes	No	No
2c	No	Yes	Yes	Yes	Yes	No	Yes
3a	No	No	No	No	No	No	No
3b	No	Yes	Yes	Yes	Yes	No	No
3c	No	Yes	Yes	Yes	Yes	No	Yes
4b	No	Yes	Yes	Yes	Yes	No	No

**Fig. 4:**
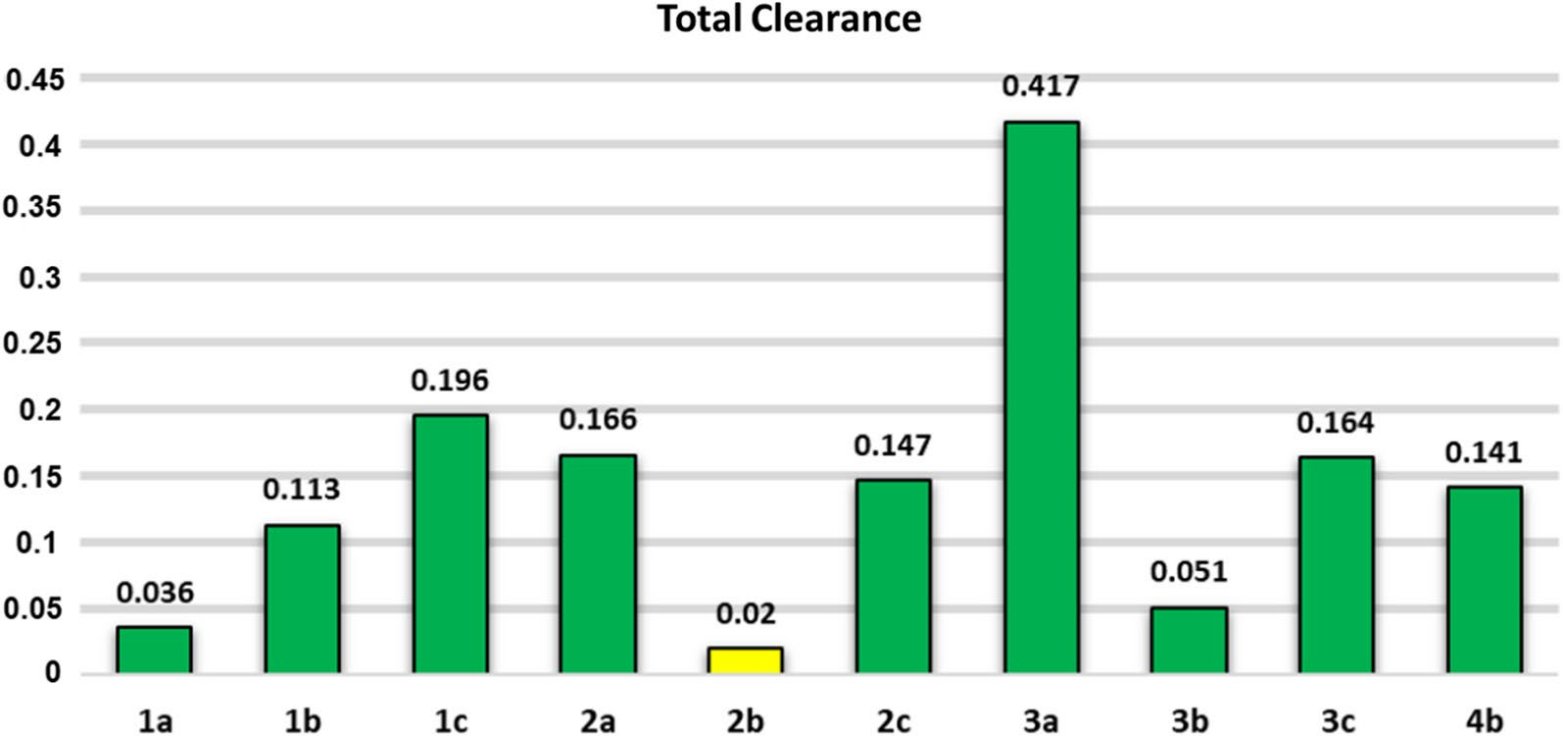
The histogram of total renal clearance profiles of the chalcones with its OCT2-dependence clearance, green block = OCT2-independent and yellow block = OCT2-dependent

Total clearance describes the compound’s rate while being removed from the body [36]. One of the main renal uptake transporters to remove the drug from the blood is the OCT2 transporter. It plays a pivotal role in the removal and renal clearance of mostly cationic drugs as well as endogenous compounds [37]. Inhibition of OCT2 (such as by cimetidine) elevates OCT2-dependent renal clearance drugs, hence altering pharmacokinetics and pharmacodynamics profiles. Compound **3a** is predicted to be the fastest compound excreted from the body due to its highest total clearance. In contrast, compound **2b** is the slowest compound to be removed from the body due to its lowest total clearance. All chalcones have low total clearance (log Cl < 0.763), yet, it is generally desirable to develop a drug for oral administration without a high dosage regimen [38]. One chalcone, **2b**, is predicted to act as renal OCT2 substrate that might lead to undesirable side effects. Figure 4 illustrates the total clearance of all chalcones that reflects their rate to be eliminated from the body system.

### Neuraminidase assay

The chalcone derivatives have been examined for their inhibitions toward the activity of H1N1 and H5N1 influenza neuraminidase. The first screening using 100 µg/ml of the sample’s concentration showed that five compounds exhibited 0–10% inhibition toward H1N1 NA, while only one compound showed activity in the same range against H5N1. Furthermore, three compounds exhibited 10–50% inhibition toward H1N1, while five compounds demonstrated inhibition against H5N1 at the same inhibition percentage. In the H5N1 NA inhibition assay, two compounds (**1b** and **2c**) were inactive. Interestingly, two compounds (**1c** and **2b**) showed more than 50% inhibition to both H5N1 and H1N1. Therefore, they were selected to be further studied on their IC_50_ as well as CC_50_. Table 8 presents the assay results of ten chalcone compounds compared to vanillin as the positive control.

**Table 8:** The assay result of ten chalcone compounds with their R side chains as depicted in Fig. 5 (100 µg/mL) against H5N1 and H1N1 NA compared to vanillin as the positive control

Compounds	R_1_	R_2_	R_3_	R_4_	% Inhibition ± SE	
					H1N1 NA	H5N1 NA
**1a**	H	OH	H	F	21 ± 7	23 ± 11
**2a**	H	OH	H	NO_2_	16 ± 2	21 ± 2
**3a**	H	OH	H	COOH	19 ± 2	27 ± 0
**1b**	H	*O-*isopropyl	H	F	5 ± 8	0 ± 3
**2b**	H	*O-*cyclopentyl	H	F	69 ± 2	70 ± 1
**3b**	H	*O-*benzyl	H	F	2 ± 4	18 ± 3
**4b**	H	*O-*cyclopentyl	H	NO_2_	5 ± 5	15 ± 3
**1c**	OCH_3_	OH	H	*O-*butyl	83 ± 1	82 ± 3
**2c**	OCH_3_	OH	OCH_3_	*O-*benzyl	1 ± 4	− 2 ± 16
**3c**	H	OH	OCH_3_	*O-*benzyl	2 ± 4	7 ± 8
Vanillin	–	–	–	–	86 ± 2	84 ± 4

**Fig. 5:**
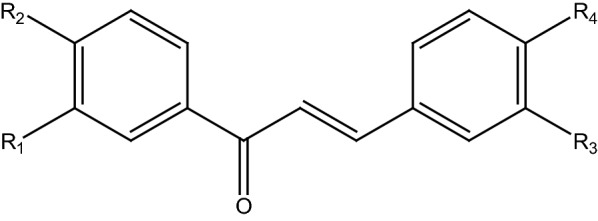
The chalcone backbone scaffold of 10 chalcone derivatives

**Fig. 6:**
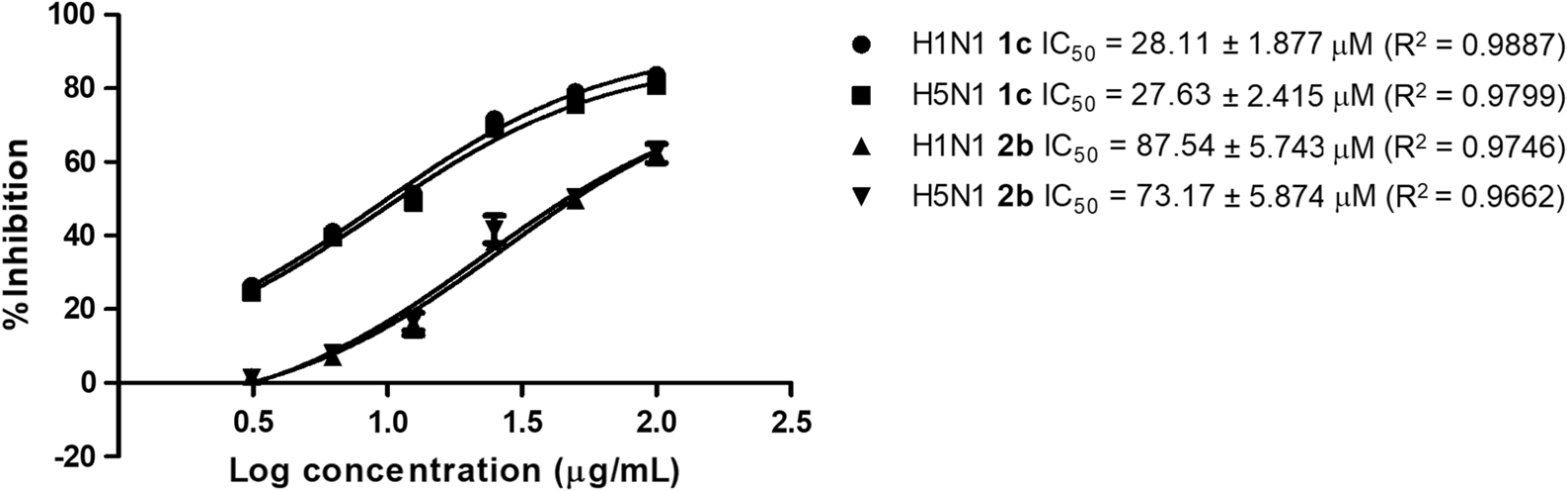
The drug–dose depending curve of **1c** and **2b** in inhibiting H1N1 and H5N1 NA

The IC_50_ of **1c** was then estimated as 28.11 µM and 27.63 µM for H1N1 and H5N1, respectively. Compound **2b** also demonstrated similar % inhibition toward H1N1 and H5N1 NA with its IC_50_ 87.54 µM and 73.17 µM, respectively. This is quite interesting because the compound showed similar IC_50_ when it was applied to both H5N1 and H1N1 NA. Figure 6 presents the drug-dose depending curve of **1c** and **2b** in inhibiting the H1N1 and H5N1 NA.

### Cytotoxicity assay

**Fig. 7:**
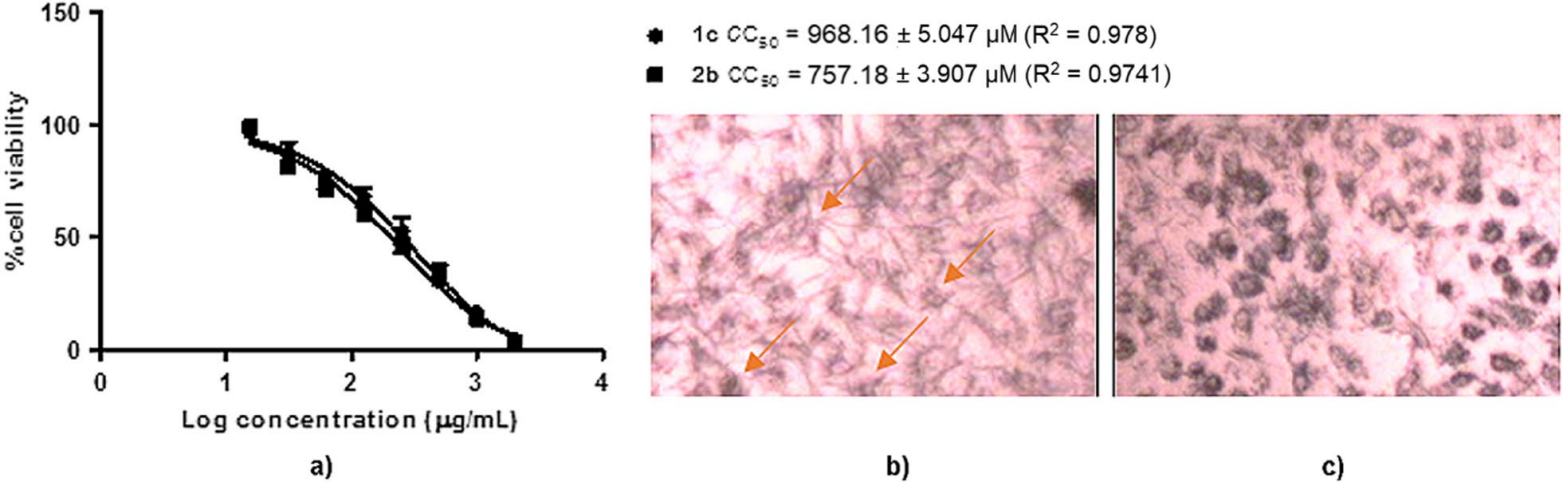
The cytotoxicity profiles of **1c** and **2b** plotting **a** log concentration of vs % Vero cell viability of **1c** and **2b**, whereas **b** is the Vero cell imaging without any compound exposure (negative control), and **c** with **1c** treatment to the cells. The orange arrows indicate the formazan crystal formation which is higher in **1c** absence than its presence, accordingly

In the cytotoxicity assay results (Fig. 7), **1c** showed a high concentration to inhibit the Vero cell proliferation with CC_50_ 968.16 µM. The safety index of this compound calculated with the safety index (SI) values was 34.44 and 35.69 for H1N1 and H5N1 NA, respectively. In addition, **2b** showed CC_50_ 757.18 µM with the SI values = 8.65 and 10.35 for H1N1 and H5N1, respectively. The graph plotting log concentration of **1c** and **2b** vs % cell viability against Vero cell in the cytotoxicity studies is presented in Fig. 7a. Furthermore, the cell imaging in Fig. 7b exhibited the cell proliferation survival, when there was no treatment to the cell, which was indicated by the formation of formazan crystals upon MTT reaction. Formazan crystal indicates the ability of the cell to express NADPH-dependent oxidoreductase to reduce the MTT, which leads to cell proliferation. In contrast, the formazan crystal formation will be reduced along with the treatment of **1c** and **2b** on the Vero cell leading to their cytotoxicity properties (Fig. 7a).

## Discussion

The 2009 H1N1 NA is one of several 2009 pandemic influenza virus mutants in which the I223 changed to R223 (I223R). This mutation results in shrinkage of the enzyme’s active site affecting the binding of NA inhibitor [39]. Another mutant is H274Y (H1N1), which was also reported to be resistant to oseltamivir as confirmed in about 20% isolates from humans in Europe [40, 41]. NA’s active site is composed of eight functional residues (ARG118, ASP151, ARG152, ARG224, GLU276, ARG292, ARG371 and TYR406) and surrounded by 11 framework residues (GLU119, ARG156, TRP178, SER179, ASP198, ILE222, GLU227, HIS274, GLU277, ASN294 and GLU425) [42].

Structurally, chalcone consists of one aromatic ring extended by a predominantly *trans*-configuration of *α,β*-unsaturated carbonyl. The relatively short carbon–carbon distance between *α,β*-unsaturated alongside the relatively long C–C distances is 1.326 Å and 1.46 Å, which is consistent with a localized double bond in the enone unit in this structure [43]. The chain next to the carbonyl group is usually prolonged by either alkyl or aryl moieties with various functional groups being attached. The conjugated double bond could make this type of compound less flexible. However, the C=O bond can present as either S-*cis* or S-*trans* conformation with respect to the vinylic double bond due to the free rotation along with the single bond between C-carbonylic and Cα. This, therefore, could increase the conformation stability of such compounds during a dynamic environment [44].

The combination of in silico and in vitro studies has suggested a new binding mode for chalcone compounds into N1 NA. The active site of NA is decorated by a relatively small pocket surrounded by some sub-pockets determining the binding region of such enzyme with a particularly striking feature in the catalytic domain referred to as the ‘150 loop’ [39, 45]. The shikimic acid scaffold has been classically patterned for NA inhibitors due to its capability to form a stable chair conformation transition state of the non-aromatic six-member ring with the rather flat oxonium cation, and thereby arrange the position of important residues to interact with the shikimic binding groups [45]. Taking this into consideration, some aromatic compounds have been devised to mimic this transition state leading to their inhibition against NA [46, 47].

**Fig. 8:**
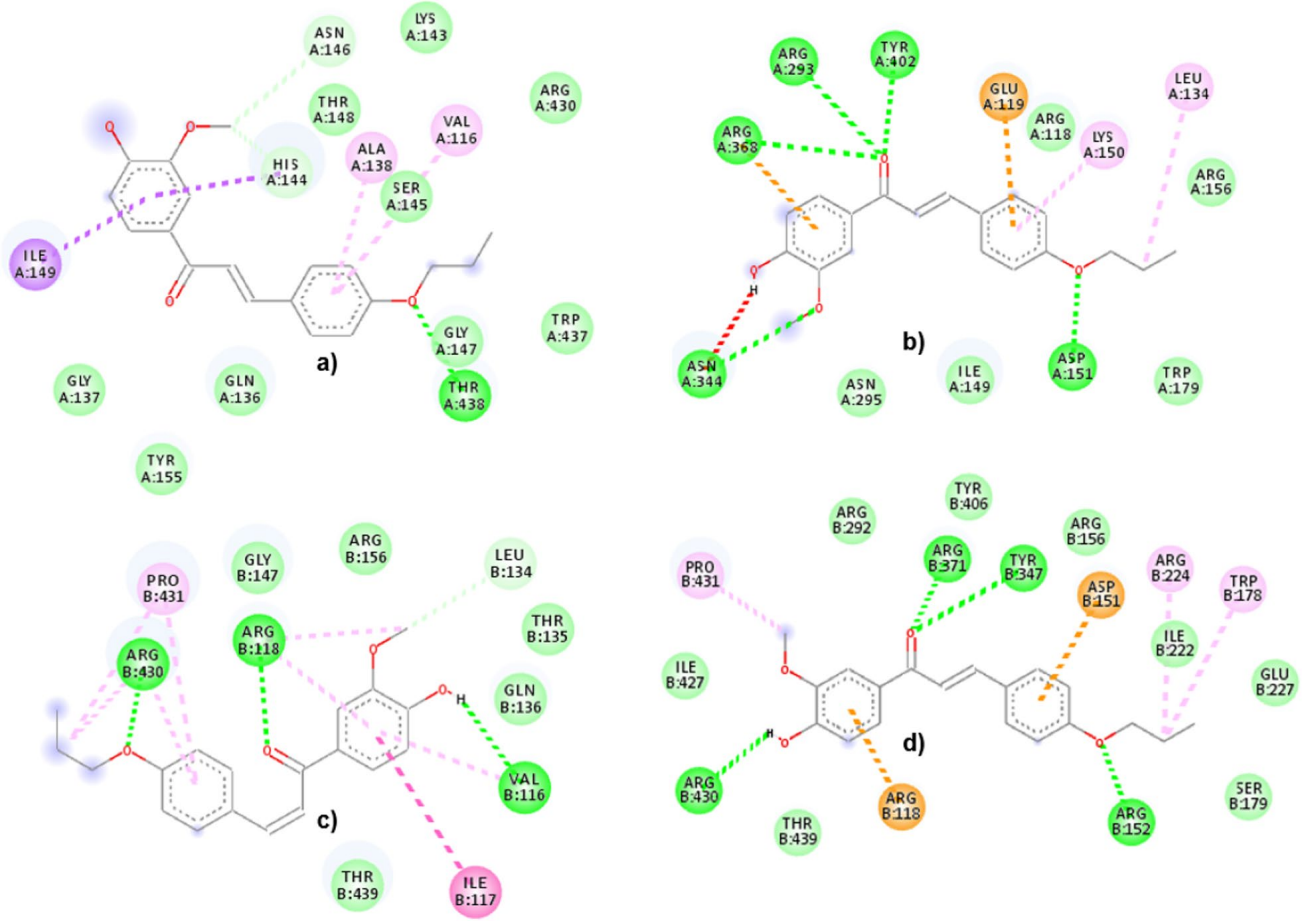
The molecular interactions of **1c** with **a** non-catalytic H1N1, **b** catalytic H1N1, **c** non-catalytic H5N1, and **d** catalytic H5N1 NA sites. The green, pale green, purple, magenta, pink, cyan, and orange represent H-bond, vdW, pi-sigma, amide pi-stacked, pi-alkyl, pi-halogen, and pi-cation/anion, respectively

**Fig. 9:**
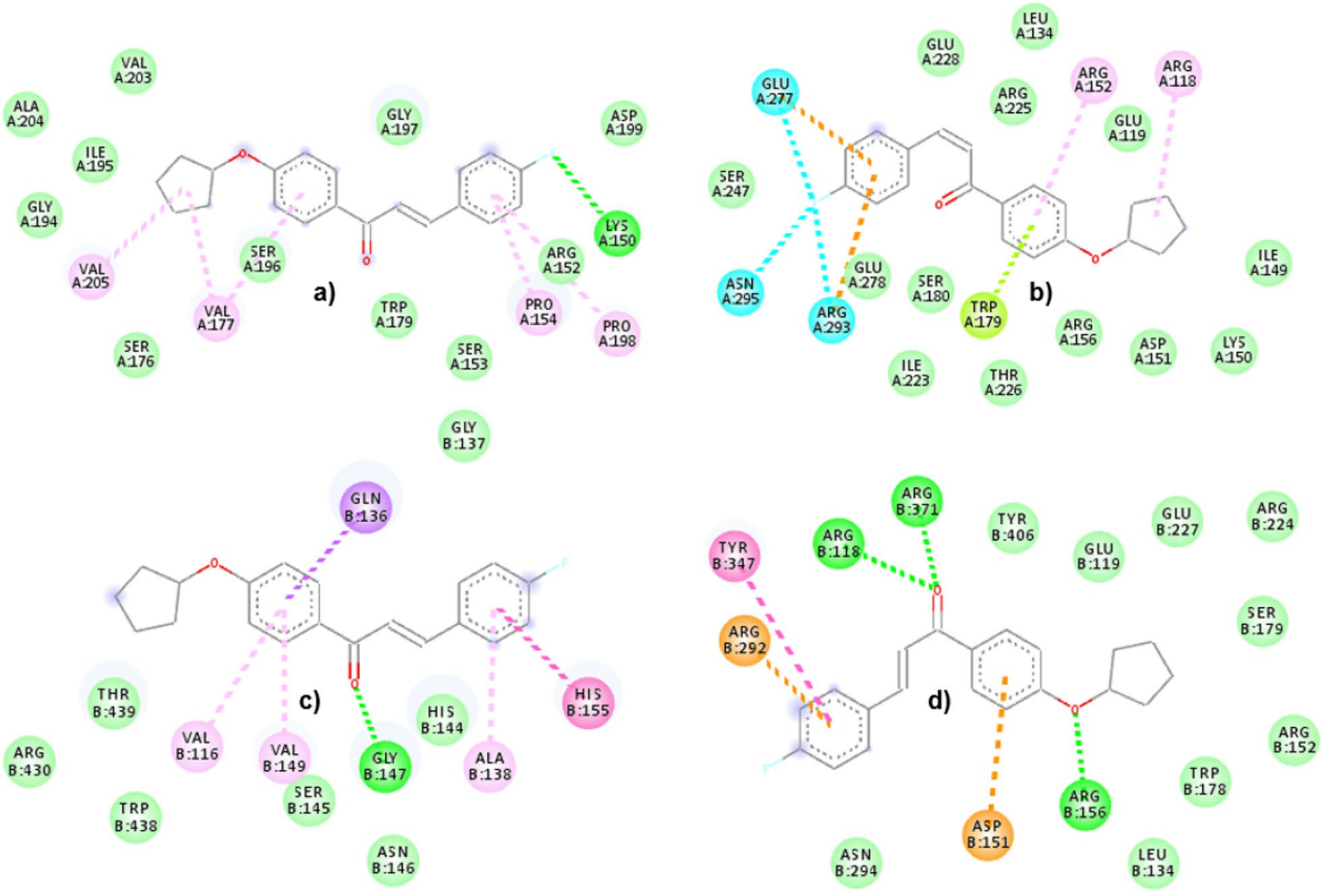
The molecular interactions of **2b** with **a** non-catalytic H1N1, **b** catalytic H1N1, **c** non-catalytic H5N1, and **d** catalytic H5N1 NA sites. The green, pale green, purple, magenta, pink, cyan, yellowish-green and orange represent H-bond, vdW, pi-sigma, amide pi-stacked, pi-alkyl, pi-halogen, pi-lone pair electrons, and pi-cation/anion, respectively

In the present study, two chalcones (**1c** and **2b**) have been observed to have the best in vitro results compared to the other 8 chalcones. Figures 8 and 9 illustrate the molecular interactions of **1c** and **2b**, respectively, while binding into the H1N1 (non- and catalytic sites) and H5N1 (non- and catalytic sites). During the binding into the non-catalytic site of H1N1 NA, **1c** interacts via H-bond with only THR438. The other interactions are vdW interactions with ASN146 and HIS144; amide pi-stacked with HIS144 and ILE149; and pi-alkyl interactions with VAL116, ALA138 and SER145. In contrast, the binding to the catalytic site of H1N1 NA shows that **1c** interacts with ASP151, ARG293, ASN344, ARG368, and TYR402 via H-bonds, whereas pi-alkyl and alkyl-alkyl interactions are also observed with LYS150 and LEU134, respectively. In addition, pi-anion interaction is observed with GLU119. Compound **1c** interacts with the non-catalytic site of H5N1 NA via H-bonds with VAL116, ARG118, and ARG430; amide pi-stacked with ILE117; and pi-alkyl with VAL116, ARG118, ARG430 and PRO431. Furthermore, in its catalytic site, the interactions are observed via H-bonds with ARG152, ARG371, TYR347, and ARG430; alkyl interactions with TRP178, ARG224, and PRO431; pi-cation as well as pi-cation interactions are observed with ARG118 and ASP151, respectively.

The second best compound (**2b**) also interacts via H-bond with the non-catalytic site of H1N1 NA with LYS150; pi-alkyl with PRO154, VAL177, PRO198 and VAL205. In contrast, the interactions with the catalytic site via H-bonds are absent. However, the remaining interactions of **2b** are observed with ARG118 and ARG152 via pi-alkyl interactions; GLU277, ARG293 and ASN295 via pi-halogen interactions; with TRP179 via pi-lone pair electron interaction. The non-catalytic site of H5N1 NA shows interactions with chalcones via H-bonds with GLY147 and pi-alkyl with VAL116, ALA138, VAL149. In addition, amide pi-stacked with HIS155 is also observed. In the H5N1 NA catalytic site, chalcones make interactions via H-bonds with ARG118, ARG156 and ARG371. Furthermore, there are also amide pi-stacked interaction with TYR347, as well as pi-cation/anion with ASP151 and ARG292, respectively.

The possibility of chalcone to interact with the active site of NA is only in one aromatic ring augmented by the binding group such as OH or OCH_3_. The *α,β*-unsaturated chain most likely protruded outside of the active pocket. Although the ΔG_bind_ of chalcone in the active site is considered acceptable, it is inconsistent with the in vitro results. Compound **1c** has relatively higher ΔG_bind_ in both NAs’ active sites than other compounds with poor inhibition against NAs. However, the in vitro results showed that **1c** is the most active compared to others; indicating a poor correlation between in vitro and in silico studies. In contrast, although **2c** is inactive against NAs in vitro, the ΔG_bind_ in both NA’s active sites is considered fair.

Notably, compounds with –NO_2_ group (**2a** and **4b**) show the lowest binding energy in either non-catalytic or catalytic sites for both H1N1 and H5N1 NAs. However, the in vitro results demonstrated a low percentage of inhibition toward both NAs. Nitro group is the strongest electron-withdrawing, which reactively interacts with atoms having an electron-donating group to form H-bond as well as electronic interactions [48]. This false-positive results could be due to the stability of the –NO_2_ group, which is easily reduced by the acidic pH turning into an amine group (nitro reduction) [49], which is measured in pH 6.5 in this MUNANA system. This conversion could make the **2b** and **4b** lose their interactions as suggested by the docking study, leading to their low inhibition against NA. The inconsistent results of **2b** and **3b** are not fully understood as well. Therefore, we suggest that a molecular dynamics simulation is performed to elucidate this phenomenon in future studies. On the other hand, there are inconsistent results between docking and in vitro results when the chalcones were docked into either non-catalytic and catalytic sites of H5N1. Therefore, it is recommended to study the kinetics of NA inhibition for the two most active compounds (**1c** and **2b**) to confirm whether the mode of inhibition is competitive or non-competitive.

The kinetic assay can be carried out using methods such as Biacore or Isothermal Titration Calorimetry (ITC). Biacore measures the real-time binding association and dissociation rates using Surface Plasmon Resonance (SPR). In this method, the protein is immobilised onto a biosensor surface while the drug ligand is continuously flowing across the biosensor surface, where it binds to the immobilised receptor. The binding is measured by the kinetic association and dissociation rates (k_a_/k_d_) for several different ligand concentrations [50].

The second method is ITC, which measures the heat transfer during binding that enables accurate determination of binding constants (K_D_), reaction stoichiometry (n), enthalpy (∆H) and entropy (ΔS). This provides a complete thermodynamic profile of the molecular interaction. This deeper understanding of structure–function relationships enables more confident decision making in hit selection and lead optimization [51].

In this present study, an array of in silico predictions has been performed on the chalcones including Lipinski Rule, mutagenicity, toxicity and pharmacokinetic profiles. The chalcones have diverse functional groups conferring the distinction of their drug-like properties, mutagenicity, toxicity, and pharmacokinetics, which will contribute to their overall therapeutic effects. Given that the two compounds (**1c** and **2b**) are active in the in vitro study, we are motivated to understand the possibility of these compounds to be considered as lead candidates for optimization. They have good Lipinski Rule profiles by meeting the requirements of MW, log P, number of HBD-HBA, number of rotatable bonds and the surface area. Both compounds are also not responsive against the AMES test describing their non-mutagenic potency. Unfortunately, **2b** is predicted to have a low tolerance in the maximum dose. In addition, both compounds are responsive toward hERG II as inhibitors and having an acute toxic dose of around 200 mg/kg, but irresponsive to hERG I as an inhibitor, not toxic in chronic ingestion, and neither hepatotoxic nor skin sensitized. In environmental damage, both are non-toxic to *T. pyriformis* as well as minnow species.

These two compounds are insoluble in water. Thus, consideration should be given for a suitable delivery system. They have good in Caco2 permeability, intestinal human absorption and skin permeability; reflecting their good absorption profile either in oral or topical administration. Both compounds have potencies to inhibit the protein carrier during absorption but neither of them acts as the protein substrate. In the distribution profiles, both compounds bind tightly in the plasma protein, which could reduce the therapeutic dose. Furthermore, the BBB and CNS permeability should be taken into account. Interaction with other drugs should be evaluated as well, as these two compounds are predicted to act as the CYP3A4 substrate, CYP1A2, CYP2C19, and CYP2C9 inhibitors. In contrast, they are irresponsive toward CYP2D6 and CYP3A4. The excretion of **1c** is faster than **2b** since the total clearance of **1c** is higher than **2b** in addition to the potential of this compound as Renal OCT2’s substrate. Overall, **1c** meets the requirements of drug-like properties at approximately 62%, whereas **2b** is at 58%. These percentages are above 50%, therefore, these two compounds should be given the opportunity to be optimized and developed further as drug candidates. This conclusion is supported by the in vitro cytotoxicity study against normal cell lines confirming that both compounds have a good safety index augmenting their potency as the potential H5N1 and H1N1 NA inhibitors.

## Materials and methods

### Chemistry

**Fig. 10:**
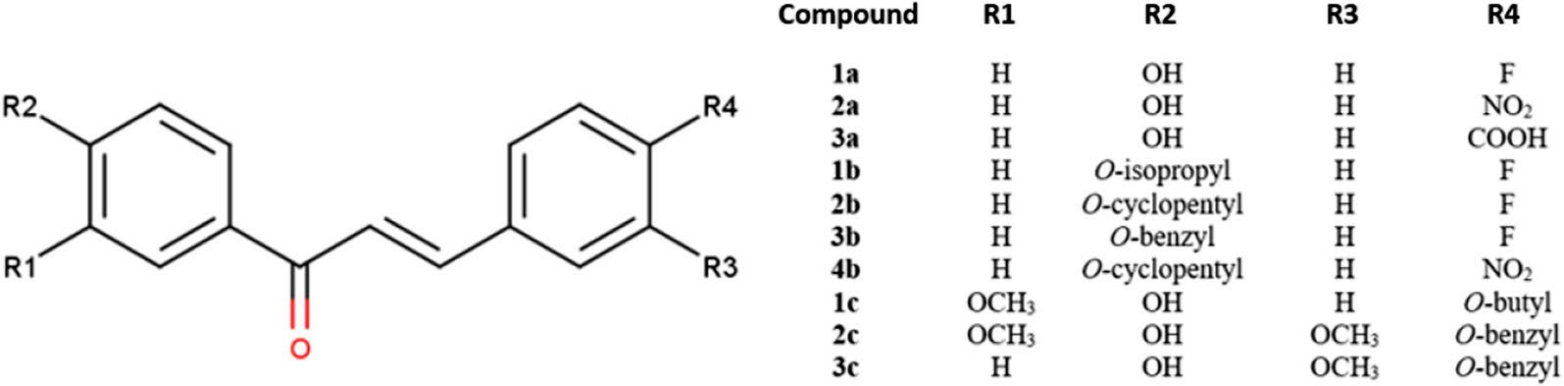
The structure of 10 chalcone derivatives (**1a**–**4b**) with different set of functional groups

The chalcone compounds (Fig. 10) were synthesized using the established method in our laboratory [21, 52, 53]. We scale up the production of the compounds and confirm their purity using a thin-layer chromatographic method and melting point test with the data of published compounds as the reference.

### Molecular docking

The proteins used are the resistant I223R NA mutant of H1N1 2009 pandemic influenza virus (PDB ID 4B7M) [39] and the wild type H5N1 NA (PDB ID 2HU0) [54]. The protein structure was processed using AutoDockTools 1.5.6 (www.autodock.scripps.edu) with the ligand separated from the protein. The grid for docking to the non-catalytic site of H1N1 NA and H5N1 NA was set to 94 × 72 × 96 and 80 × 80 × 80 with its spacing set to 0.375 Å. The center of mass of the ligand was set to x = 26.852, y = − 32.014, z = − 1.019 for H1N1 NA, and x = − 7.07, y = 28.242, z = 107.729 for H5N1 NA. Genetic Algorithm was chosen for docking calculation in AutoDock 4.2.3. The searching parameters were set to the default values (Population size = 150, maximum number of evals = 2,500,000, maximum of generations = 27,000, maximum number of top individuals that automatically survives = 1). The number of GA run was set to 100. Docking parameters such as random number generation, energy parameters, and step size were also set to the default values. The results were analyzed by checking the RMSD values, ligand–protein interactions, free energy of binding (FEB) as well as the number of conformations that exist in a population cluster [55]. For the subsequent molecular docking, the chalcone structure derivatives were sketched and energetically optimized using Hyperchem Professional version 8.0 (www.hyper.com) with MM + force field and Polak-Ribiere (Conjugate Gradient). The visualization of ligand–protein interaction was conducted using Biovia Discovery Studio 2016 (www.accelrys.com).

The docking of compounds to the catalytic site of NA was carried out by using PDB 2HU0 (H5N1 NA in complex with oseltamivir) [54] as well as PDB 6HP0 (H1N1 NA in complex with oseltamivir triazole) [56] with the same default protocol being used in PDB 4B7M, except for the number of points and its center of mass. For 6HP0, the number of points is 40 × 40 × 40 with the center of mass (x = 43.641; y = 0.53; z = 20.263). For 2HU0, the number of points is 40 × 40 × 40 with the center of mass (x = 1.763; y = 19.33; z = 108.34).

### Lipinski rule of five

The Lipinski Rule profiles were individually predicted by inputting SMILES string, which is automatically done by the server. The value of molecular weight (MW), log P, the number of hydrogen bond donors (HBD), the number of hydrogen bond acceptors (HBA), the number of rotatable bonds and the surface area were observed and then tabulated.

### Mutagenicity and toxicity studies

Using the same protocol as in “Lipinski rule of five” section, the mutagenic potency of the ligands was represented by the AMES test results. Other parameters such as maximum tolerated dose (human) (hMTD), hERG I inhibitor, hERG II inhibitor, oral rat acute toxicity (log LD_50_), oral rat chronic toxicity (log LOAEL), hepatotoxicity, skin sensitization, *T. pyriformis* toxicity, and minnow toxicity represented the toxicity properties of the ligands.

### Pharmacokinetics study

Using the same protocol in 4.3, the ADME (absorption, distribution, metabolism, and excretion) profiles of the ligands were predicted. Subsequently, the absorption is influenced by water solubility, Caco2 permeability, skin permeability, P-glycoprotein substrate, P-glycoprotein I inhibitor, and P-glycoprotein II inhibitor instead of human gastrointestinal absorption. The distribution is represented by VDss (human), fraction unbound (human), blood–brain barrier (BBB) permeability, and central nervous system (CNS) permeability. The metabolism is represented by the CYP2D6 substrate, CYP3A4 substrate, CYP1A2 inhibitor, CYP2C19 inhibitor, CYP2C9 inhibitor, CYP2D6 inhibitor, and CYP3A4 inhibitor. Lastly, the excretion is represented by the total clearance and renal OCT2 substrate. The Lipinski Rule, mutagenicity, toxicity and pharmacokinetic prediction were carried out using pkCSM online tool (http://biosig.unimelb.edu.au/pkcsm/prediction) [28].

### Neuraminidase assay

The H1N1 NA assay followed the general procedure of Fluorometric Neuraminidase Assay [57]. The H1N1 NA (A/California/04/2009) and H5N1 NA (A/Anhui/1/2005) enzymes were purchased from Sinobio. The fixed concentrations of H1N1 NA (0.3 u/mL) and MUNANA (100 mM) were optimized employing the previously described method. Vanillin was used as the positive control inhibitors [58], and the H1N1 neuraminidase assay was prepared by mixing an assay buffer, tested samples (at concentrations 100 μg/mL in 1% of DMSO-Buffer), and a constant 0.3 unit/mL of neuraminidase which were pre-incubated at 37 °C for 30 min with 200 rpm. After the addition of 100 μM substrates, the reaction assays were incubated at 37 °C for 60 min with 200 rpm. To stop the reaction, 100 μl of glycine stop solution was added. The assays were carried out in triplicate. The assay protocol for H5N1 inhibition assay is similar to that of H1N1 but the concentrations being used were 0.15 u/mL, and 50 µM for the enzyme and substrate, respectively. A series of concentrations were prepared for those demonstrating > 50% of enzyme inhibition to calculate the IC_50_. The fluorescence intensity of NANA was measured by Modulus Microplate Reader with a UV optical kit at λ 340/440 nm. The drug-dose dependent curve and its statistical analysis (95% confident interval) were generated using GraphPad Prism 5.0 (https://graphpad-prism.software.informer.com/5.0/).

### Cytotoxicity assay

The cytotoxicity of each compound on Vero cells was determined using MTT assay. Vero cell is a non-tumorigenic cell from the kidney tissue of African green monkeys [59]. Cells will proliferate by expressing the NADPH-dependent oxidoreductase in mitochondria that reduces the MTT reagent into the reduction state of formazan crystal [60]. Cells (1 × 10^4^/well) were seeded in 96-well flat-bottomed plates and incubated with each sample at various concentrations for 24 h. Compound solutions were prepared in the following concentrations: 10, 20, 40, 80, 160, 320, 640 and 1280 µg/mL. 30 μL of MTT solution (5 mg/mL in PBS) was added to each well and the plate was incubated at 37 °C for another 4 h. Then, the medium was discarded and 150 µl of DMSO was added to dissolve the formazan crystals. The absorbance of each sample was read at 595 nm using a microplate reader. Results were expressed as a percentage of cell viability with respect to untreated control cells (as 100%) [61]. The drug-dose dependent curve and its statistical analysis (95% confident interval) were generated using GraphPad Prism 5.0 (https://graphpad-prism.software.informer.com/5.0/).

## Conclusions

Computational studies using molecular docking, ADME-Tox prediction and in vitro study, including their H5N1 and mutant H1N1 NA inhibitory activity and cytotoxicity towards Vero cells of the 10 synthesized chalcone derivatives have been employed to predict and evaluate the inhibitory mechanism and the safety of these chalcone derivatives. In conclusion, **1c** ((*E*)-3-(4-butoxyphenyl)-1-(4-hydroxy-3-methoxyphenyl)prop-2-en-1-one) and **2b** ((*E*)-1-(3-(cyclopentyloxy)phenyl)-3-(4-fluorophenyl)prop-2-en-1-one) have potencies to be developed as anti-influenza drugs by inhibiting H5N1 and H1N1 NA in the non-catalytic site.

## Supplementary Information

**Additional file 1: Figure S1.** The control docking results of **a** oseltamivir triazole into H1N1 catalytic site and **b** oseltamivir into H5N1 catalytic site.
