# Supplementary material for: A study on catalytic and non-catalytic sites of H5N1 and H1N1 neuraminidase as the target for chalcone inhibitors
Source: Appl Biol Chem. 2021 Sep 17;64(1):69. doi: 10.1186/s13765-021-00639-w (PMC8445792; doi:10.1186/s13765-021-00639-w)
Supplement: Supplementary file 1 — Additional file 1: Figure S1. The control docking results of a oseltamivir triazole into H1N1 catalytic site and b oseltamivir into H5N1 catalytic site. [file 13765_2021_639_MOESM1_ESM.docx]

Additional Materials

A Study on Catalytic and Non-Catalytic Sites of H5N1 and H1N1 Neuraminidase as the Target for Chalcone Inhibitors

Pandu Hariyono^1^_,_ Jasvidianto Chriza Kotta^1^, Christophorus Fideluno Adhipandito^1,2^, Eko Aprilianto^1,3^, Evan Julian Candaya^1,4^, Habibah A. Wahab^5^_,_ Maywan Hariono^1^*

^1^ Faculty of Pharmacy, Sanata Dharma University, Campus III, Paingan, Maguwoharjo, Depok, Sleman 55282, Yogyakarta, Indonesia

^2^ Faculty of Biomedical Engineering, Taipei Medical University, Wuxing Street No. 250, Xinyi District, Taipei City 110, Taiwan

^3^ PT. Dankos Farma, Jalan Rawagatel Blok IIIS Kav 35-39, Jatinegara, Cakung, Jakarta Timur 13930, DKI Jakarta, Indonesia

^4^ Apotek Kimia Farma Sempidi Unit Bisnis Nusa Dua, Jalan Raya Sempidi No. 12, Mengwi, Badung 80351, Bali, Indonesia

^5^ Pharmaceutical Technology Department, School of Pharmaceutical Sciences, Universiti Sains Malaysia, Minden 11800, Pulau Pinang, Malaysia

*****Correspondence: [mhariono@usd.ac.id](mailto:mhariono@usd.ac.id) ; Tel.: +62-895-0628-6901


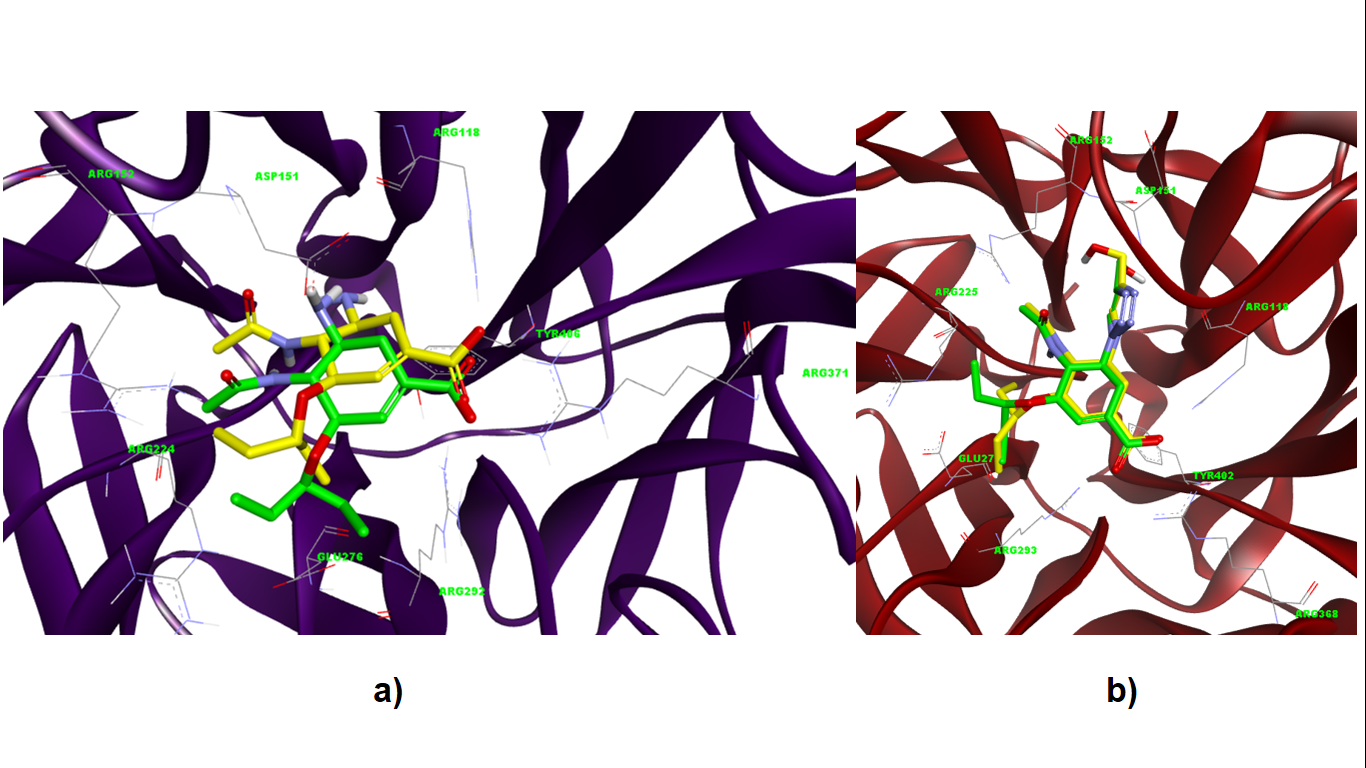


Figure S1. The control docking results of a) oseltamivir triazole into H1N1 catalytic site and b) oseltamivir into H5N1 catalytic site.
